# Supplementary material for: Comparison of Methods for Incorporating Related Data When Developing Clinical Prediction Models: A Simulation Study
Source: Biom J. 2026 Aug 27;68(5):e70166. doi: 10.1002/bimj.70166 (PMC13520826; doi:10.1002/bimj.70166)
Supplement: Supplementary file 2 [file BIMJ-68-e70166-s001.zip › third submission code review - R2/reproducibility_supplement/results/Plots/validation_mterics_plot_low_degree_casemix_SC_ID5_TS0.25_DS4_model15.pdf]

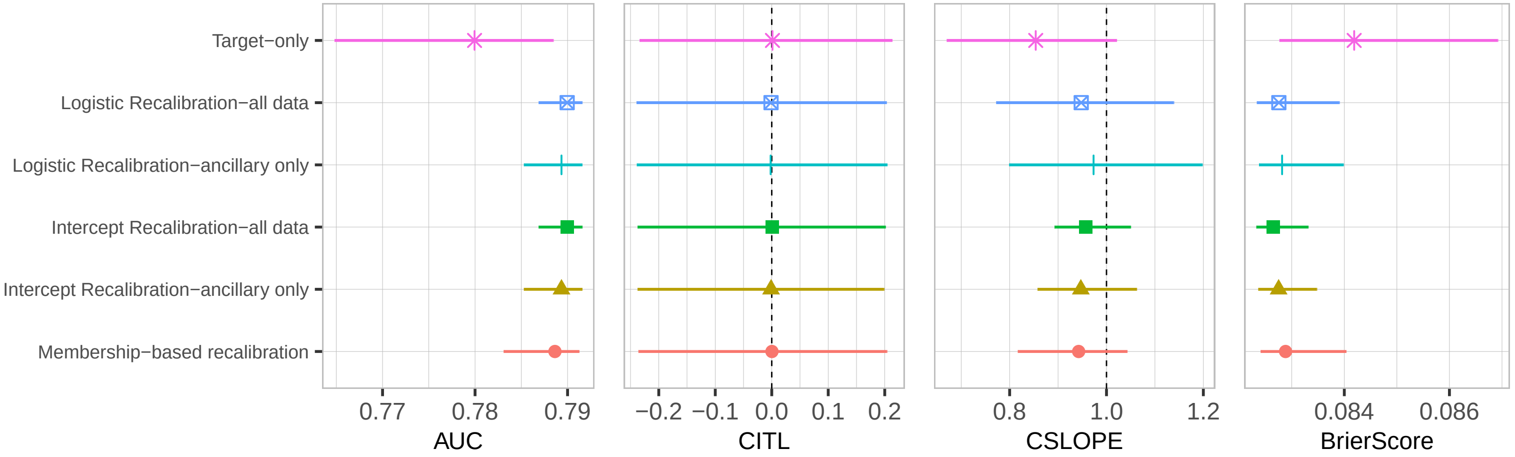

Target−only
Logistic Recalibration−all data
Logistic Recalibration−ancillary only
Intercept Recalibration−all data
Intercept Recalibration−ancillary only
Membership−based recalibration
0.77
0.78
0.79
AUC
−0.2
−0.1
0.0
0.1
0.2
CITL
0.8
1.0
1.2
CSLOPE
0.084
0.086
BrierScore
